# Biomechanical analysis of adjacent segments after correction surgery for adult idiopathic scoliosis: a finite element analysis

**DOI:** 10.1038/s41598-024-63113-9

**Published:** 2024-06-08

**Authors:** Dong-hai Wang, Dan-ni Wu, Da-qi Xin, Qin Shi, Wen-xuan Wang, Wen-hua Xing, Hui-lin Yang

**Affiliations:** 1 Department of Orthopedics, The First Affiliated Hospital of Soochow University, Orthopedic Institute of Soochow University, 188 Shizi Road, Suzhou, 215006 Jiangsu People’s Republic of China; 2 School of Kinesiology, Shanghai University of Sport, Research Building 412, 200 Hengren Road, Shanghai, 200438 People’s Republic of China; 3 Department of Orthopedics, The Second Affiliated Hospital of Inner Mongolia Medical University, Orthopedic Institute of Inner Mongolia Autonomous Region, 59 Horqin South Road, Hohhot, 010090 Inner Mongolia People’s Republic of China; 4 Department of Orthopedics, The Children’s Hospital of Soochow University, 92 Zhongnan Street, Suzhou, 215025 Jiangsu People’s Republic of China

**Keywords:** Adult idiopathic scoliosis, Adjacent segment degeneration, Stress distribution, Finite element analysis, Posterior corrective surgery, Biomedical engineering, Medical research

## Abstract

The biomechanical aspects of adjacent segment degeneration after Adult Idiopathic Scoliosis (AdIS) corrective surgery involving postoperative changes in motion and stress of adjacent segments have yet to be investigated. The objective of this study was to evaluate the biomechanical effects of corrective surgery on adjacent segments in adult idiopathic scoliosis by finite element analysis. Based on computed tomography data of the consecutive spine from T1–S1 of a 28-year-old male patient with adult idiopathic scoliosis, a three-dimensional finite element model was established to simulate the biomechanics. Two posterior long-segment fixation and fusion operations were designed: Strategy A, pedicle screws implanted in all segments of both sides, and Strategy B, alternate screws instrumentation on both sides. The range of motion (ROM), Maximum von Mises stress value of intervertebral disc (IVD), and Maximum von Mises stress of the facet joint (FJ) at the fixation adjacent segment were calculated and compared with data of the preoperative AdIS model. Corrective surgery decreased the IVD on the adjacent segments, increased the FJ on the adjacent segments, and decreased the ROM of the adjacent segments. A greater decrease of Maximum von Mises stress was observed on the distal adjacent segment compared with the proximal adjacent segment. The decrease of Maximum von Mises stress and increment of Maximum von Mises stress on adjacent FJ in strategy B was greater than that in strategy A. Under the six operation modes, the change of the Maximum von Mises stress on the adjacent IVD and FJ was significant. The decrease in ROM in the proximal adjacent segment was greater than that of the distal adjacent segment, and the decrease of ROM in strategy A was greater than that in strategy B. This study clarified the biomechanical characteristics of adjacent segments after AdIS corrective surgery, and further biomechanical analysis of two different posterior pedicle screw placement schemes by finite element method. Our study provides a theoretical basis for the pathogenesis, prevention, and treatment of adjacent segment degeneration after corrective surgery for AdIS.

## Introduction

Adult idiopathic scoliosis (AdIS) involves the curvature of the spine due to unknown causes in patients over 18 years of age. Its prevalence varies from 1.4%–20%^1^. Generally, untreated patients with adolescent idiopathic scoliosis progress to AdIS with ageing^2^. Many patients with AdIS are asymptomatic in adolescence, and hence, do not seek surgical intervention to correct the deformity. However, if the spinal deformity is left untreated, the symptoms may worsen and impair pulmonary function due to curve progression. Corrective surgery in well-defined indications and individualized surgical procedures reportedly provide good prognosis for AdIS^3^. However, studies have reported adjacent segment degeneration associated with spinal fusion surgery^4^. Although the incidence of adjacent segment degeneration is not very high, its long‐term effects are significant^5^. The latest studies attribute the underlying mechanics of adjacent segment degeneration to its pathogenesis, although the pathogenesis remains unclear^6,21^. Hence, the mechanical aspects of adjacent segment degeneration involving postoperative changes in kinematics (motion and displacement) and/or kinetics (stress and force) of the adjacent segments need to be further investigated.

Experiments involving animal models and cadaver specimens are carried out routinely to investigate the biomechanics of the spine. AdIS is a complex, three-dimensional (3D) deformity of the spine involving bending of the coronal plane, change of the sagittal plane contour, and rotation of the transverse plane. Concurrently, local structural deformities develop in pedicles, spinous and transverse processes, vertebral bodies, and intervertebral discs^7^. It is not feasible to replicate such a complex structure by engineering an animal model of AdIS, and cadaver specimens of AdIS are difficult to obtain. Hence, in silico investigations are most suitable to best understand the pathophysiology.

Finite element analysis (FEA) is a well stablished mathematical technique for simulating physical phenomena by discretizing the domain into finite elements. FEA is used to study spine biomechanics and parameters internal to the spine and connective soft tissues, which are difficult to capture using experimental approaches. The finite element model (FEM) can reproduce a complex spinal deformity using reverse engineering software. Few researchers have performed a biomechanical analysis of spinal deformities using FEA. Zhang et al.^8^ used computed tomography (CT) data to construct a 3D-FEM of the lumbar spine in adolescent idiopathic scoliosis to explore the biomechanical changes of the lumbar spine segment of idiopathic scoliosis under different loads. Chen et al.^9^ applied FEA to study the biomechanics of implants and implant-bone interface for different pedicle screw placement strategies in treatment of Lenke 1 AdIS. Similarly, Kumaran et al.^10^ used FEA to study adjacent segment degeneration in transforaminal lumbar interbody fusion by comparing open and minimally invasive surgeries to explore the biomechanical mechanism of adjacent segment degeneration. However, no studies have elaborated on the biomechanical principles of adjacent segment degeneration after corrective surgery for AdIS.

The current study simulated posterior corrective surgeries on AdIS by the FEM. FEA was performed to explore the influences of correction processes on the range of motion (ROM), intradiscal pressure, and facet stress of adjacent unfused segments. At present, there are two pedicle screw placement strategies for AdIS: (1) All Vertebral Pedicle Screw Strategy, and (2) Interval Vertebral Pedicle Screw Strategy. Bilateral all pedicular screw placement can obtain reliable fixation and reduce the concentration of stress on each pedicle screw. Bilateral interval pedicle screw placement can reduce the number of pedicle screws, shorten the operation time, save medical expenses, and increase the area of bone graft bed to obtain rapid and firm fusion^9^. No studies have elaborated on the biomechanical principles of adjacent segment degeneration after corrective surgery for AdIS using these two different pedicle screw placement strategies. Our study simulated these two corrective surgeries on the finite element methods model of AdIS. FEA was performed to explore the influences of different correction processes on the ROM, intradiscal pressure, and facet stress of adjacent unfused segments.

## Methods

###  Patient selection

A 28-year-old male patient with Lenke 3 AdIS was recruited for this study. The study was approved by the Ethics Review Committee of our hospital, and the patient provided written informed consent for the study. All methods were performed in accordance with the relevant guidelines and regulations.The maximum Cobb angles of the thoracic coronal plane and lumbar coronal plane were 52.61° (T8–T12) and 49.80° (L1–L5), respectively. The sagittal parameters were − 6.39° (T4–T11) and 57.56° (L1–L5). The Cobb angles of the right-bending radiographs were 36.41° (T8–T12) and 58.42° (L1–L5), and those of the left-bending radiographs were 61.10° (T8–T12) and 24.10° (L1–L5).

### Construction of the AdIS finite element model

**Figure 1:**
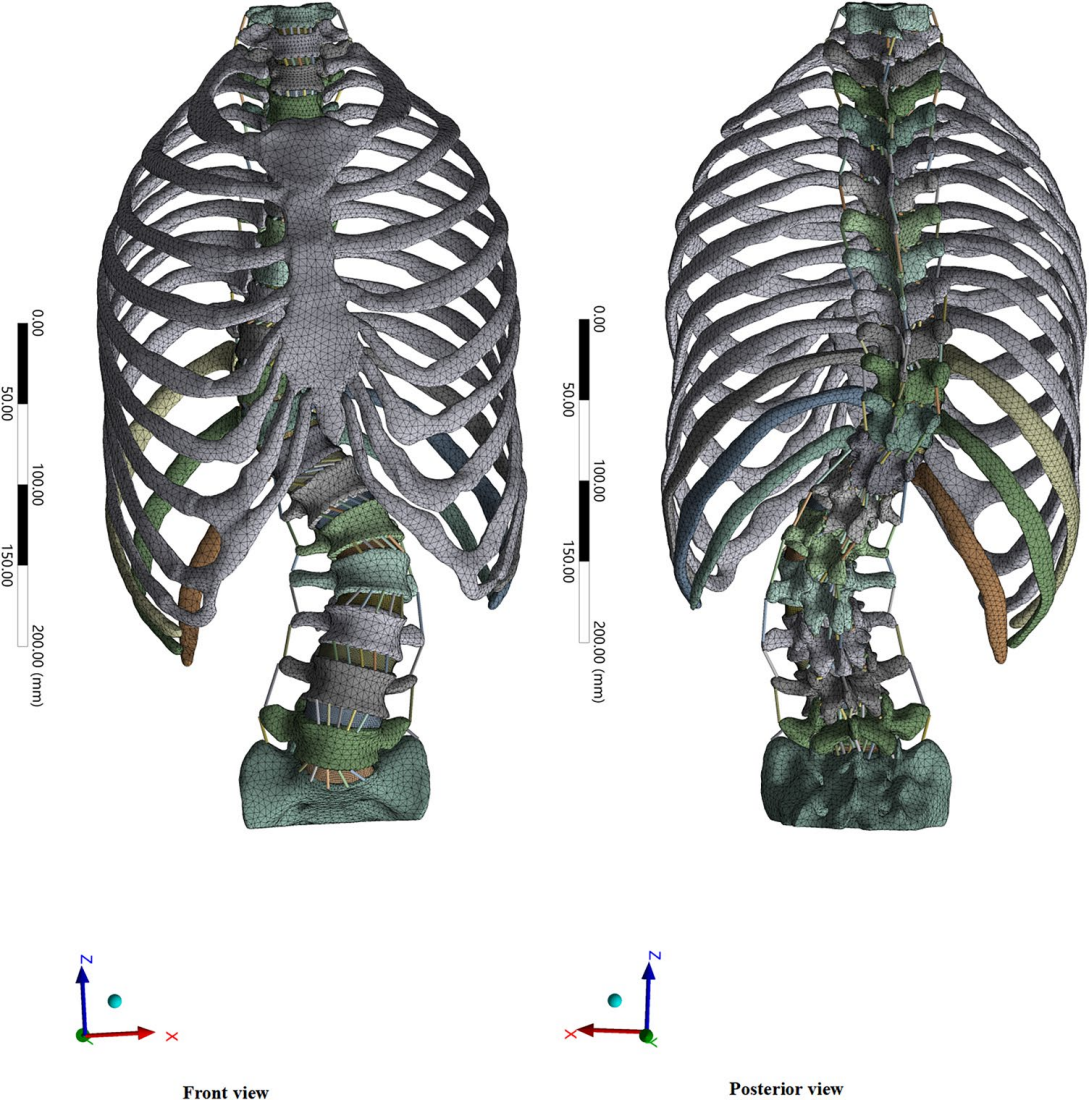
Three-dimensional finite element model of adult idiopathic scoliosis (front view and posterior view).

A 3D-FEM was constructed using a series of CT images in 0.625 mm slices from the patient. The 3D spine model was reconstructed using Mimics Innovation Suite 20.0 (Materialise, Belgium). The model was optimized by Geomagic 12.0 (Geomagic, USA) to facilitate the next biomechanical simulation. The ligaments were established in Ansys Workbench 18.0 (ANSYS, USA) using Rod elements Link 10 to simulate the ligaments between the vertebrae. The FEM consisted of 18 vertebrae, 17 intervertebral discs, seven ligaments (i.e. anterior longitudinal ligament [ALL], posterior longitudinal ligament [PLL], ligamentum flavum [LF], capsular ligament [CL], interspinous ligament [ISL], supraspinous ligament [SSL], and intertransverse ligament [ITL]), and 17 facet joints (Fig. 1). According to the physiological anatomical model of the spine, the annulus fibrosis and nucleus pulposus of the intervertebral disc will not be separated under the load; therefore, the contact settings between their faces were considered “bonded”. The facet joints will slide under a certain load, so the contact condition of the facet joints was set to “frictional”. Due to the presence of synovial fluid, the friction coefficient in this study was determined to be 0.01^8^. Cancellous bone, cortical bone, posterior elements and end plates were defined as isotropic homogeneous elastic materials^14^. The endplate was defined as a cartilaginous endplate. The cortical bone was defined as being 0.5 mm thick^12^. The intervertebral discs and facet cartilage were simulated to be nearly incompressible hyper-elastic materials^8^. The material properties of the bone, intervertebral disc, and ligament (Table 1) were determined using experimental literature^8,11,12,14,15^. Different materials were distinguished based on the Elastic modulus and Poisson’s ratio.

**Table 1:** Material properties of the spine finite element model.

Components	Young’s modulus (MPa)	Poisson’s ratio	Cross-section area (mm^2^)
Cortical bone	12,000	0.3	–
Cancellous bone	100	0.2	–
Annulus	4.2	0.45	–
Nucleus	1	0.49	–
Endplate	24	0.4	–
Cartilage	10	0.4	–
ALL	20	0.3	63.7
PLL	20	0.3	20
LF	19.5	0.3	40
CL	32.9	0.3	60
ISL	11.6	0.3	40
SSL	15	0.3	30
ITL	58.7	0.3	3.6
Pedicle screw	114,000	0.3	–
Cobalt-chromium rod	231,000	0.3	–

Source: Shono et al.^11^, Choi et al.^12^, Musapoor et al.^13^, Guan et al.^14^ and Zhang et al.^8^.

*ALL* anterior longitudinal ligament, *PLL* posterior longitudinal ligament, *LF* ligamentum flavum, *CL* capsular ligament, *ISL* interspinous ligament, *SSL* supraspinous ligament, *ITL* intertransverse ligament.

### Finite element model verification and validation

There is no standard verification method due to the different Cobb angles in scoliosis. The FEM of the scoliotic spine in this study was validated by simulating the supine position fulcrum bending test. Remove ribs from the finite element model, and the bolster was set to be attached to the apical vertebra. S1 was rigidly fixed, and load force on the T1 in the x-axis(horizontal axis) was adjusted to make the T1 centroid move the same distance (the horizontal distance of the T1 centroid to the central sacral vertical line [CSVL] on the x-ray film) to simulate the left and right bending tests. The verification included data in two parts: part one, measuring the scoliosis Cobb angle and comparing it with the fulcrum bending radiograph; and part two, measuring the distance from the centroid of the T1–S1 vertebral body to CSVL and comparing it with the fulcrum bending radiograph. Due to observer variability, a rule was instated determining that an Cobb angle offset between the FEM and clinical fulcrum bending was accepted within a range of 5°.

### Simulation of corrective surgery

Two posterior corrective surgeries were designed to correct the scoliosis. The screw placement segment s were from T6–S1. In Strategy A or the “All Vertebral Pedicle Screw Strategy”, pedicle screws were implanted in all segments of both sides. In Strategy B or the “Interval Vertebral Pedicle Screw Strategy”, alternate screw instrumentation was on both sides, except for the upper and lower instrumented vertebrae. Screws were implanted at an interval on the concave side and alternately implanted according to the opposite side. The joint capsule of the facet joint was released at the same time during the two posterior corrective surgeries.

**Figure 2:**
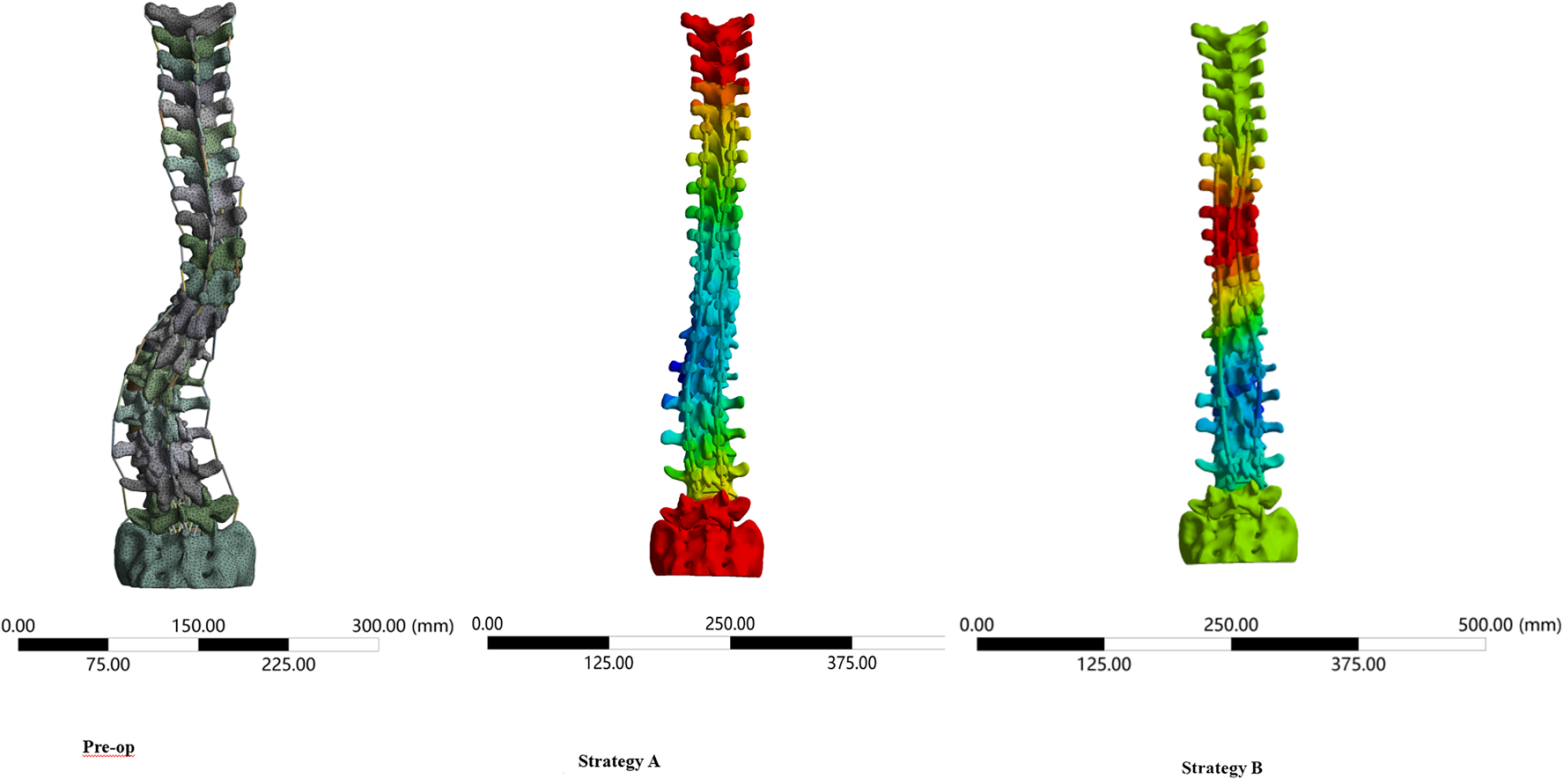
Simulation of correction surgery and correction effect. (**a**) T1-S1 spinal finite element model of adult idiopathic scoliosis (preoperative). (**b**) Correction strategy A surgery FE model: pedicle screws were implanted in all segments of both sides. (**c**) Correction strategy B surgery FE model: alternate screw instrumentation was on both sides, except for the upper and lower instrumented vertebrae.

The pedicle screw and rod were simulated by SolidWorks 2015 (Dassault, France). The dimensions of all pedicle screw were 40 × 4 mm, to simplify the model and reduce interference factors. The screw and rod models were imported into Ansys Workbench 18.0 for assembly and meshing, with a cell size of 1 mm. The screw and rod were designed as tetrahedral solid units. The degrees of freedom between the screw and vertebral body, and screw and nut interface, were limited such that the screw and nut were regarded as a whole. The screw and rod were regarded as cylindrical joints. The release of the facet joint capsule was simulated by removing the capsule ligament. The screw and rod at both ends of the whole fixation system were set as simple rotation degrees of freedom to make their movement resemble actual operation. The correction process included the rod de-rotation at the concave side and strengthening by rod placement at the convex side. After the corrective rod and pedicle screws were assembled into the FEM of the scoliotic spine, the T12 vertebra was rigidly fixed. The corrective rod was rotated 90° clockwise around the axis that connected both ends of the rod, and then the corrective rod was fixed with pedicle screws. Next, the supportive rod was implanted into the convex side and fixed with pedicle screws. Subsequently, the corrective rod was unfixed with pedicle screws so that the corrective stress can be partly distributed to the convex side (Fig. 2).

### Boundary and loading conditions

The degrees of freedom of the inferior surfaces of S1 were completely fixed in all directions. The seven different motion states of the spine (upright, flexion, extension, left and right lateral bending and rotation) were simulated under 500 N of compressive loading (the upper body is approximately 2/3 of the total body weight, 75 kg was converted to 500 N). The 7.5 N m moments of loading and bounding conditions were applied based on in vitro test loads in Yamamoto et al.^16^, and Panjabi et al.^17^ study, and FEA test loads in a study by Kim^18^. So in our study, a moment of 7.5 N m in the axial, sagittal, and frontal planes on the upper surface of the T1 vertebral body was applied to simulate flexion, extension, left and right bending, and left and right rotation. The main parameters that were investigated included spinal ROM, intradiscal pressure, and stress of the facet joint at T5–6 and L5–S1 segments.

### Ethics approval and consent to participate

This study was reviewed and approved by the Institutional Review Board of Second Affiliated Hospital of Inner Mongolia Medical University (approval number EFY20220014).

## Results

### AdIS finite element model

An AdIS (Lenke 3) FEM including the sternum, costal cartilage, ribs, thoracic vertebrae, lumbar vertebrae, sacrum, intervertebral disc, and corresponding ligaments was established. A solid 187 tetrahedral element was used for bone, link10 rod element was used for ligament, and a thickness of 0.8 mm was used for vertebral cortical bone. The whole 3D-FEM included 522,887 tetrahedral solid elements and 730 rod elements, with a total of 523,617 elements and 159,008 nodes. The vertebral body contained 137,344 elements and 221,974 nodes. The intervertebral disc contained 192,593 elements and 639,044 nodes.

### Model validation

**Figure 3:**
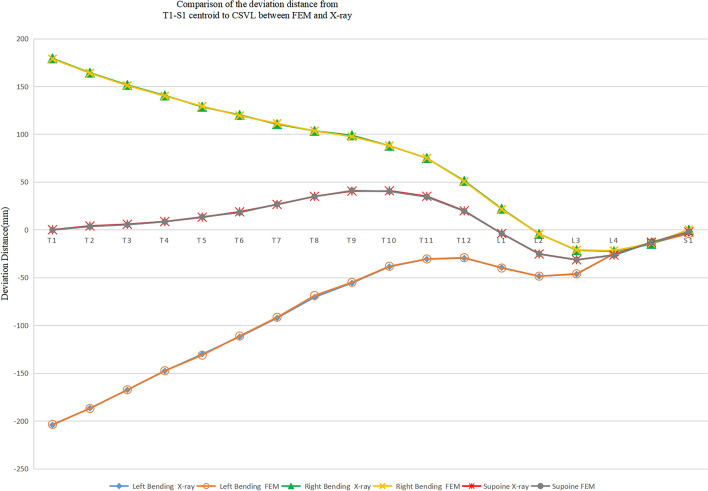
Comparison of the deviation distance from T1-S1 centroid to the CSVL between the finite element model and X-ray.

The model was deemed representative as the maximum difference in bending Cobb angles between the model and x-ray was 2.37° (Table 2). The deviation distance from the centroid of T1–S1 to CSVL of model and x-ray film was compared (Fig. 3).

**Table 2:** Comparison of Cobb angle (°) between the finite element model and x-ray.

	Thoracic Cobb angle (°) (T1–T12)		Lumbar Cobb angle (°) (L1–L5)	
	X-ray	FEM	X-ray	FEM
Left bending	52.61	54.68	21.84	22.3.0
Right bending	30	29.77	49.8	52.169

*FEM* finite element model.

### Maximum von Mises stress of adjacent segment intervertebral disc

**Figure 4:**
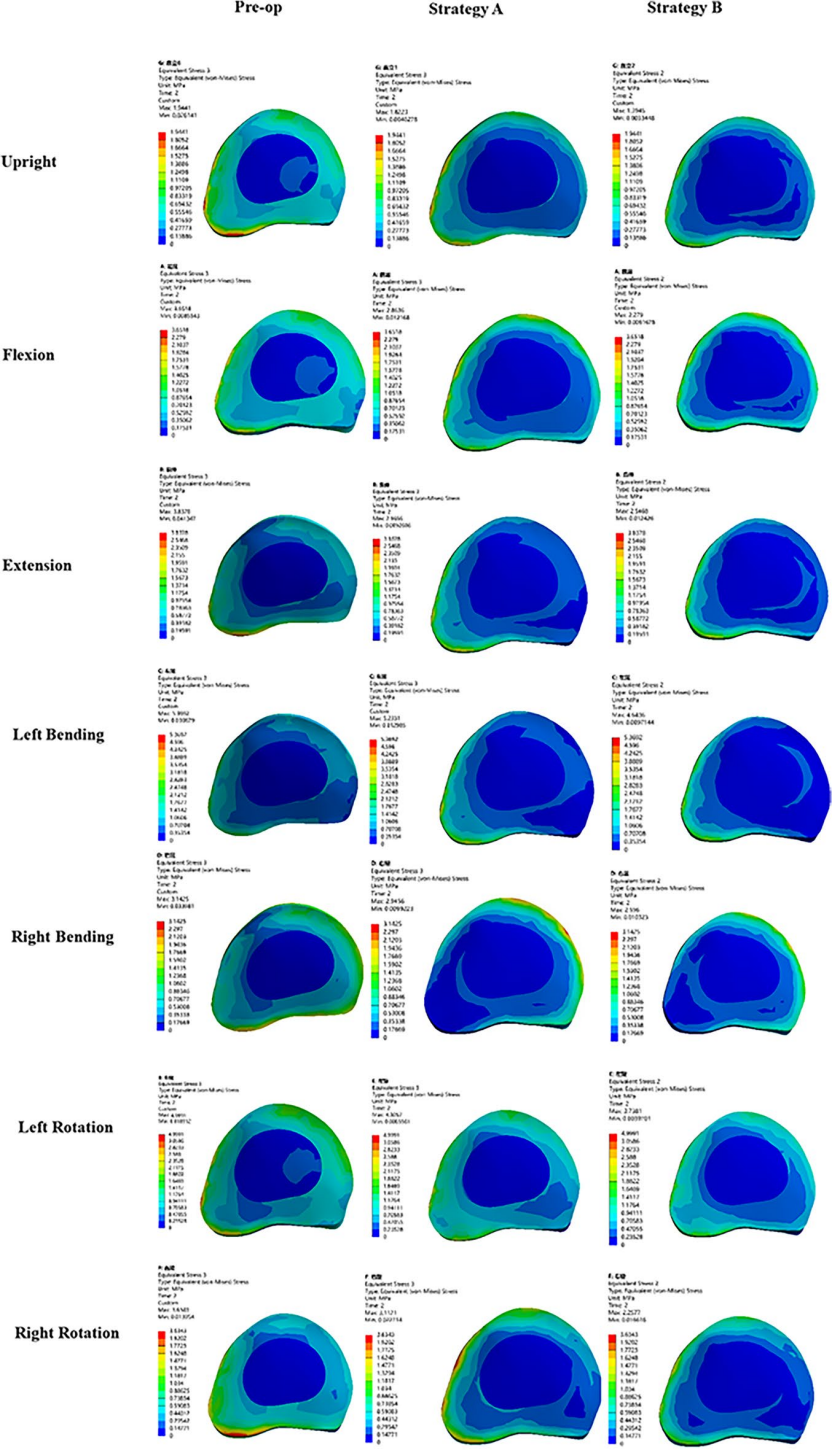
Comparison of the stress nephogram of IDP in T5–T6 in preoperative spine model, strategy A surgery model and strategy B surgery model under 7 different loading conditions (upright, flexion, extension, left bending, right bending, left rotation, right rotation).

**Figure 5:**
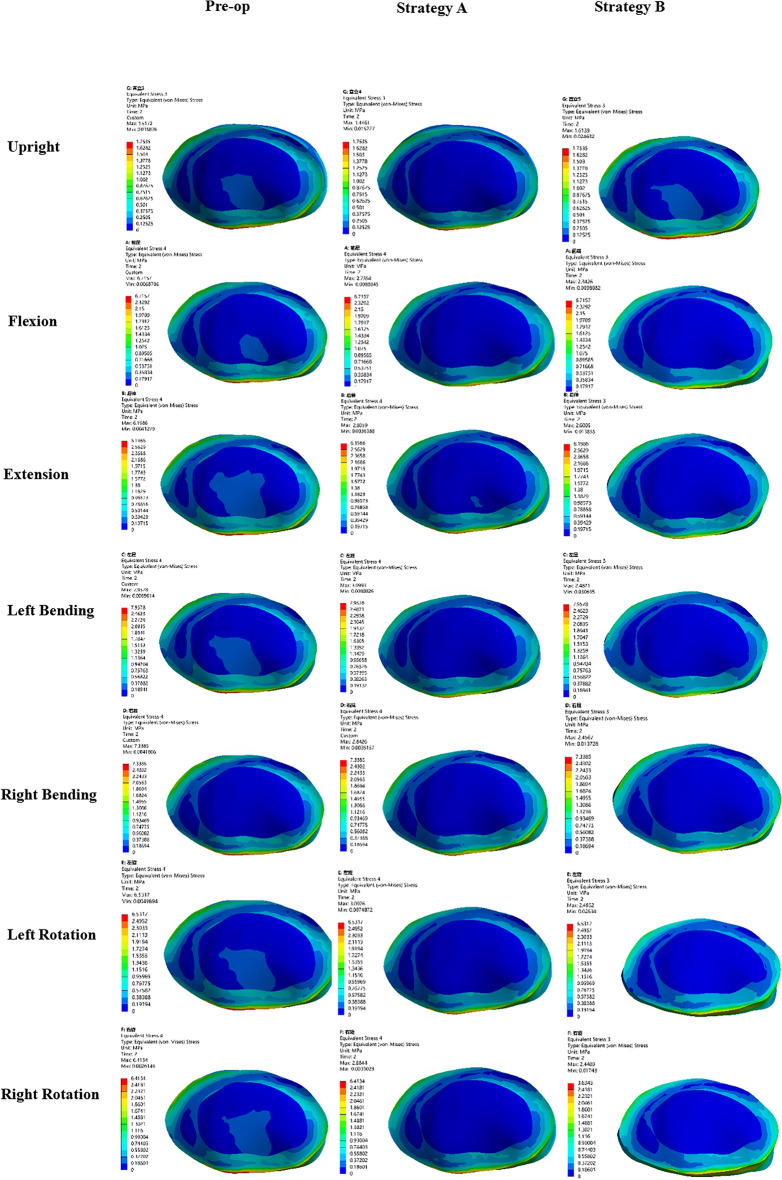
Comparison of the stress nephogram of IDP in L5–S1 in pre-operative spine model, strategy A surgery model and strategy B surgery model under 7 different loading conditions (upright, flexion, extension, left bending, right bending, left rotation, right rotation).

The post-operative stress nephogram of intervertebral discs at the proximal adjacent segment (T5–T6, Fig. 4) and distal adjacent segment (L5–S1, Fig. 5) is out of balance under different operating modes. The pre-operation stress at the proximal (T5–T6) and distal (L5–S1) adjacent intervertebral disc under upright, flexion, extension, lateral bending, and axial rotation ranged approximately from 1.94 to 5.37 MPa and 1.75 to 7.96 MPa, respectively. The post-operative stress of IVD at T5–T6 and L5–S1 under the above 7 load conditions approximately from 1.40 to 5.23 MPa and 1.40 to 3.10 MPa, respectively.

**Figure 6:**
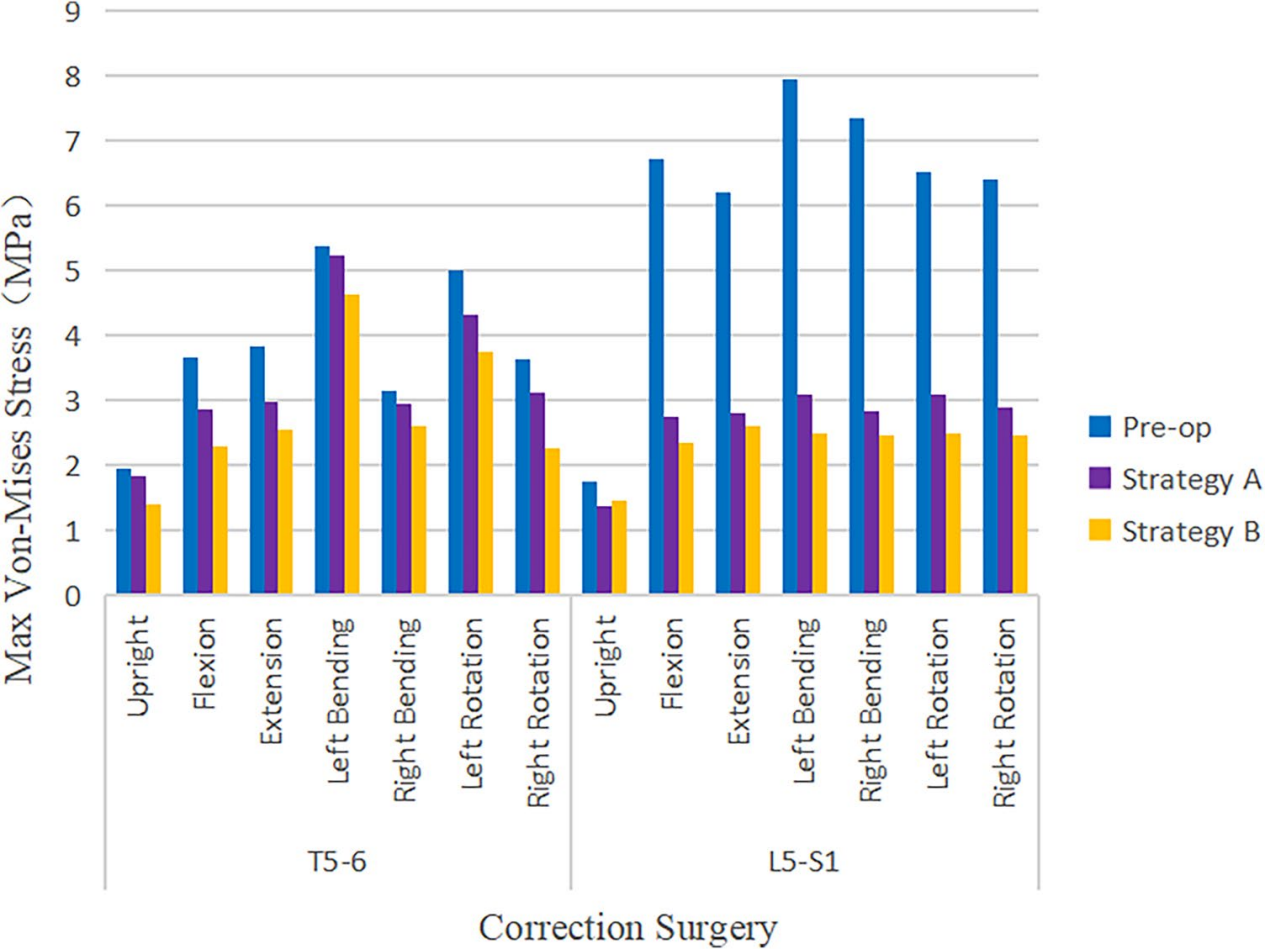
Comparison of Maximum von Mises stress value of IDP in adjacent Segments in pre-operative spine model, strategy A surgery model and strategy B surgery model under 7 different loading conditions.

**Figure 7:**
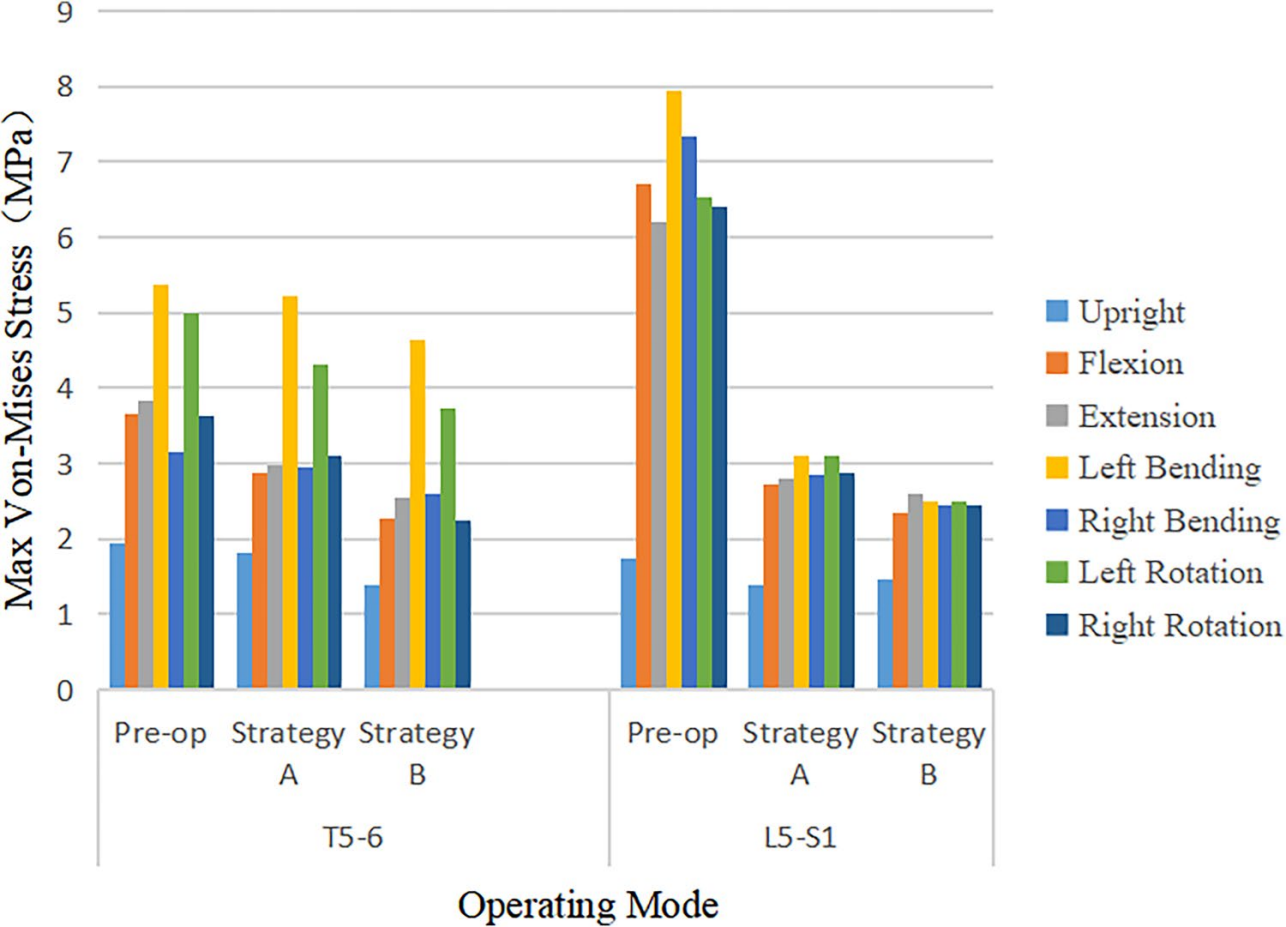
Comparison of maximum von Mises stress value of IDP in adjacent segments under 7 different loading conditions in pre-operative spine model, strategy A surgery model and strategy B surgery model.

The decrease of Maximum von Mises stress in IVD at the distal adjacent segment was greater than that at the proximal adjacent segment. The decrease of the stress in Strategy B was slightly greater than that in Strategy A (Fig. 6). The highest stress reduction on proximal adjacent intervertebral disc occurred at flexion and right rotation with the highest value of 1.37 MPa in Strategy B at the T5–T6, followed by 0.79 MPa in Strategy A, at the T5–T6. The highest stress reduction on the distal adjacent intervertebral disc occurred at lateral bending with the highest value of 5.47 MPa in Strategy B at L5–S1, followed by 4.86 MPa in Strategy A at L5–S1. There was no significant difference in the amplitude of Maximum von Mises stress reduction between the proximal adjacent segment (T5–T6) and the distal adjacent segment (L5–S1) in the upright position. There was no significant difference in the reduction of Maximum von Mises stress in the proximal adjacent segment (T5–T6) under the 7 operating modes. The Maximum von Mises stress of the intervertebral disc at the distal adjacent segment (L5–S1) decreased significantly under the six operating modes of flexion, extension, left and right lateral flexion, and left and right rotation (Fig. 7).

### Maximum von Mises Stress on adjacent segment facet joint

**Figure 8:**
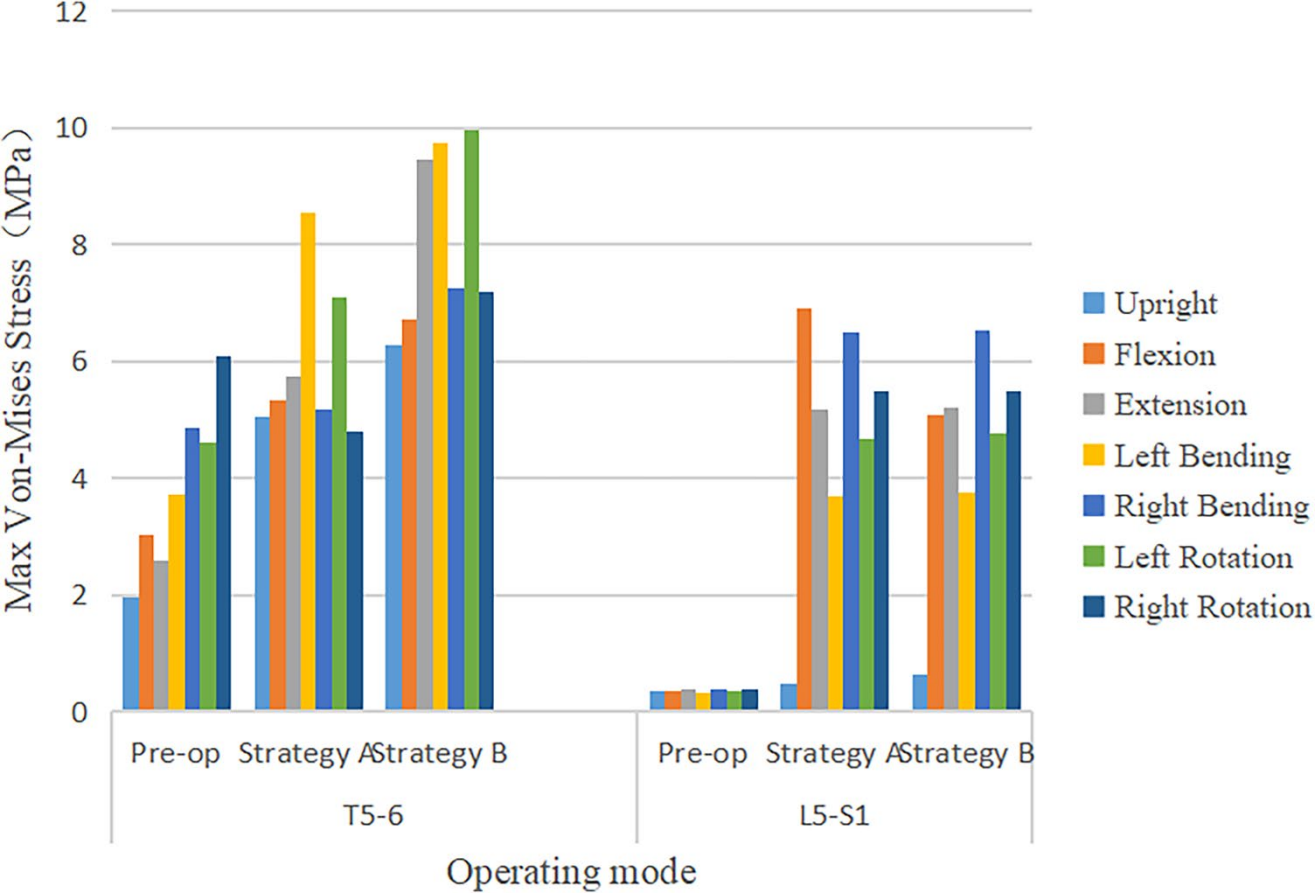
Comparison of maximum von Mises stress value of FJP in adjacent segments in pre-operative spine model, strategy A surgery model and strategy B surgery model under 7 different loading conditions.

The maximum von Mises stress of FJ at the proximal adjacent segment (T5–T6) and the distal adjacent segment (L5–S1) significantly increased under 7 operating modes (Fig. 8). The pre-operation stress value at the proximal (T5–T6) and the distal (L5–S1) adjacent facet joint under upright, flexion, extension, lateral bending, and axial rotation ranged approximately from 1.95 to 6.09 MPa and 0.34 to 0.40 MPa, respectively. The post-operative stress of FJ at the T5–T6 and L5–S1 adjacent facet joint under above 7 operating modes ranged approximately from 4.8 to 9.7 MPa and 0.5 to 6.9 MPa, respectively. The increment of Maximum von Mises stress of FJ at the distal adjacent segment outweighed the proximal adjacent segment after surgery. The post-operative stress of FJ at L5–S1 increased by 20 times.

**Figure 9:**
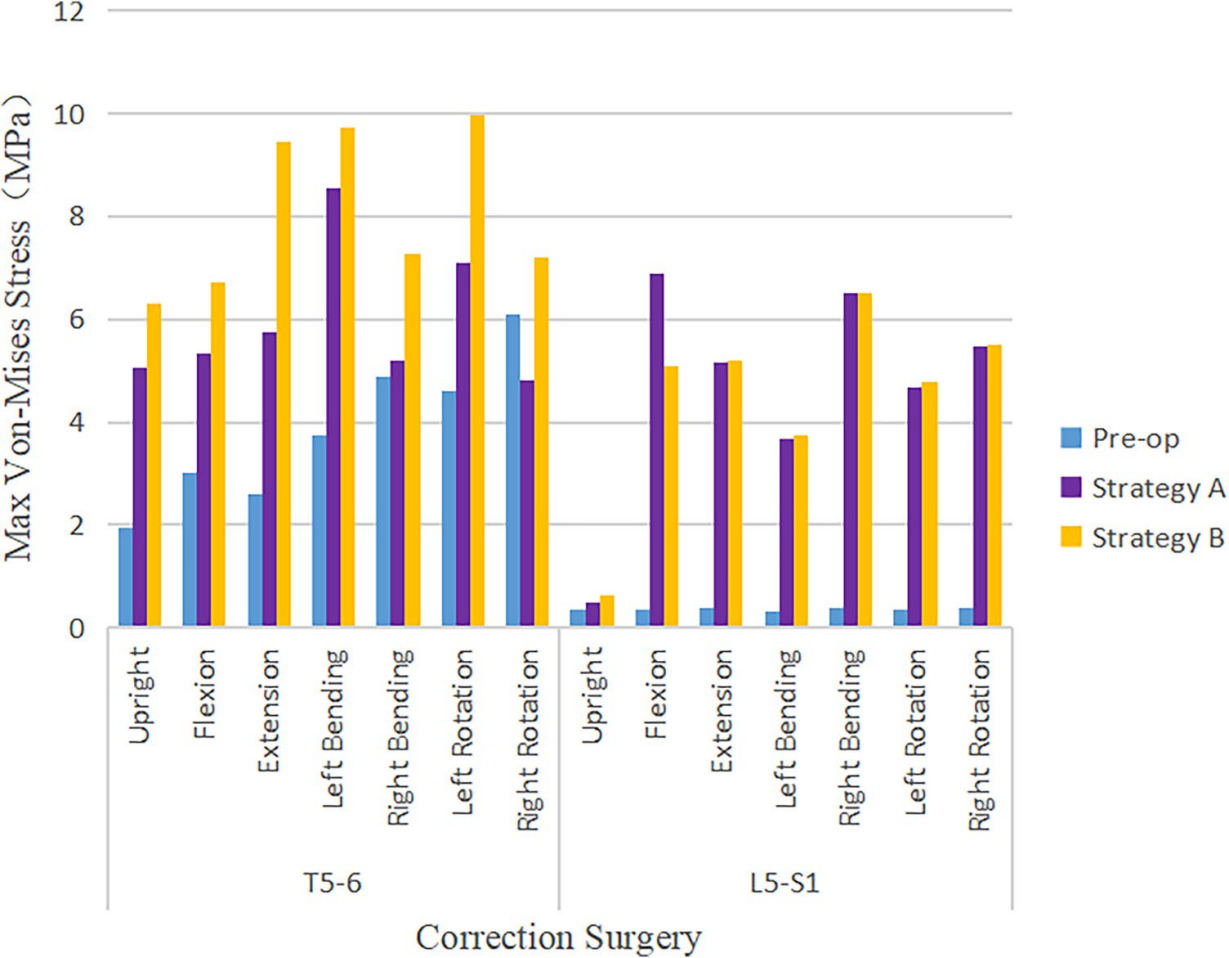
Comparison of maximum von Mises stress value of FJP in adjacent segments under 7 different loading conditions in pre-operative spine model, strategy A surgery model and strategy B surgery model.

The increment of Maximum von Mises stress on FJ at adjacent segment in Strategy B was more significant than that in Strategy A (Fig. 9). The influence of different surgical strategies on the stress of FJ in proximal adjacent segment was obvious, but it was not significant in distal adjacent segment. On the proximal adjacent segment (T5–T6), the highest increment of the stress of FJ occurred at extension and left bending with the highest value of 4.81 MPa in Strategy A and 6.86 MPa in Strategy B. The highest increment of the stress on FJ at the distal adjacent segment (L5–S1) occurred at flexion and right bending, and it increased by approximately 20 times in both Strategy A and Strategy B. There was no significant difference that Maximum von Mises stress of FJ in the distal adjacent segment (L5–S1) at the upright operating modes between pre- and post-operative. The Maximum von Mises stress of FJ in the proximal and distal adjacent segment increased significantly under the six operating modes of flexion, extension, left and right lateral flexion, and the left and right rotation.

### ROM distribution at adjacent segments

**Figure 10:**
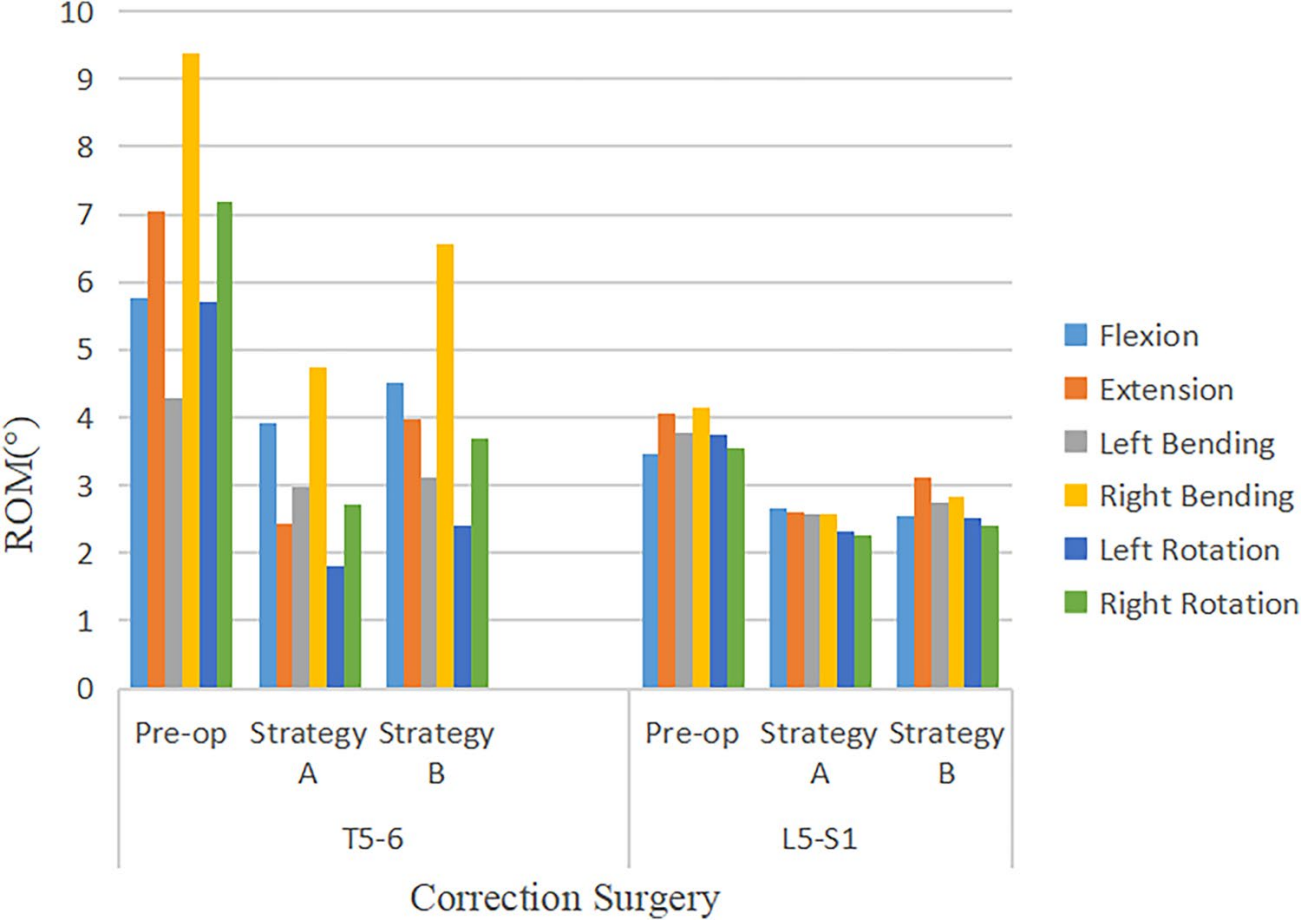
Comparison of ROM in adjacent segments in pre-operative spine model, strategy A surgery model and strategy B surgery model under 7 different loading conditions.

**Figure 11:**
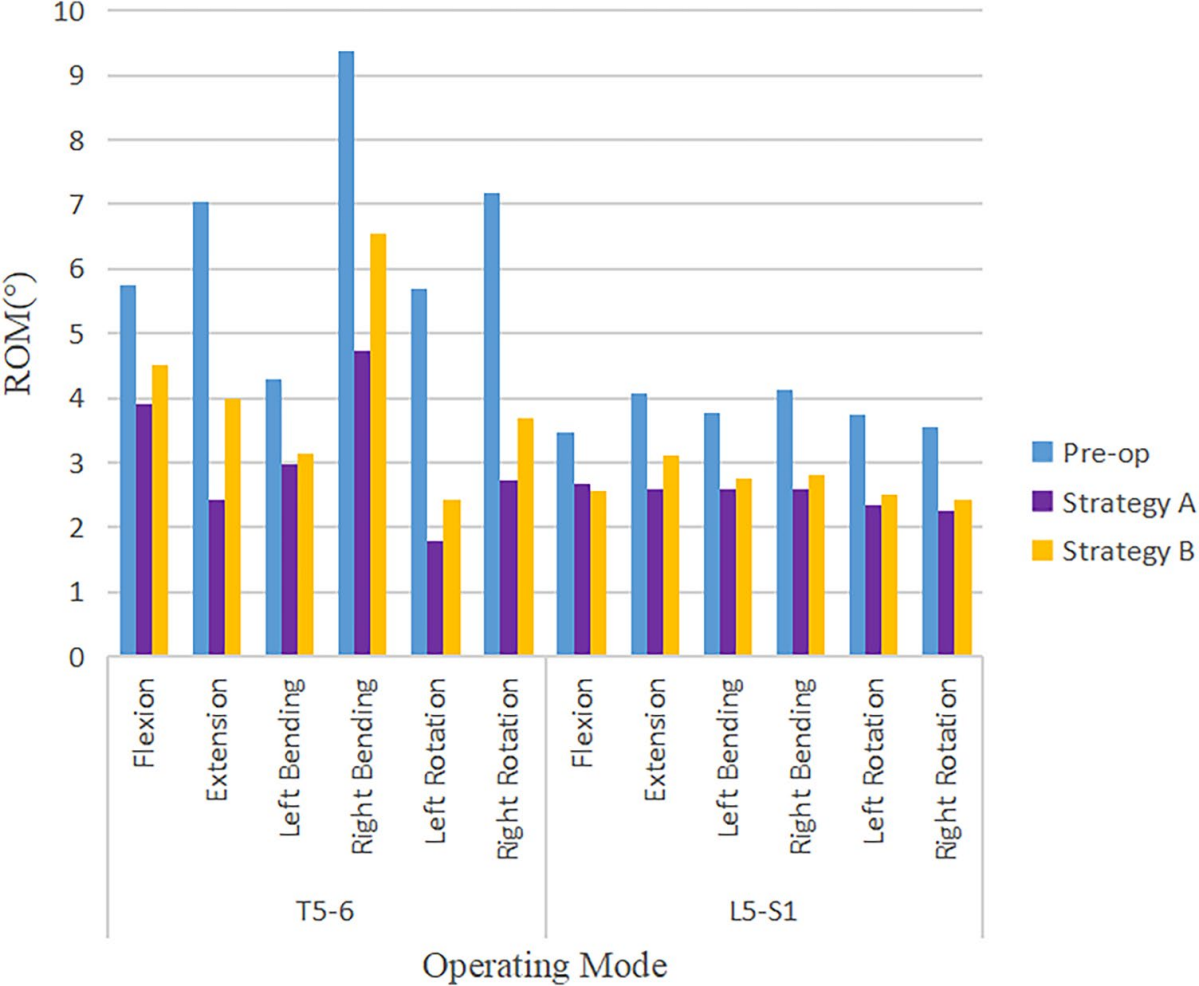
Comparison of ROM in adjacent segments under 7 different loading conditions in pre-operative spine model, strategy A surgery model and strategy B surgery model.

The ROM of flexion, extension, lateral bending, and axial rotation of the adjacent segments of the corrective surgery FEM decreased after surgery. The ROM of the proximal adjacent segment (T5–T6) and the distal adjacent segment (L5–S1) decreased under different operating modes (Fig. 10). Before the operation, the ROM at the proximal (T5–T6) and the distal (L5–S1) adjacent segment under flexion, extension, lateral bending, and axial rotation ranged approximately from 4.28° to 9.38° and 3.47° to 4.13°, respectively. For all constructs after the operation, the ROM at the proximal (T5–T6) and the distal (L5–S1) adjacent segment under flexion, extension, lateral bending, and axial rotation ranged from 1.8° to 6.55° and 2.27° to 3.11°, respectively. The decrease of ROM in the proximal adjacent segment was greater than that in the distal adjacent segment. Additionally, the decrease of ROM in Strategy A was greater than that in Strategy B (Fig. 11). The highest reduction in ROM of the proximal adjacent segment occurred at right bending and right rotation, with the highest value of 4.65° in Strategy A at the T5–T6 segment, followed by 3.48° in Strategy B at the T5–T6 segment. The highest reduction in ROM of the distal adjacent segment occurred at right bending with the highest value of 1.54° in Strategy A at the L5–S1 segment, followed by 1.31° in Strategy B at the L5–S1 segment.

## Discussion

### Overall experimental summary

Biomechanical studies on the degeneration of adjacent segments after single segment spinal fusion have been carried out with readily available cadaver specimens and animal models. It was found that the biomechanical mechanism of degeneration involves the increase of ROM, disc stress, and facet joint stress of adjacent segments after lumbar interbody fusion^19–21^. However, some researchers have proposed that interbody fusion by lordotic cage was shown to reduce the ROM and intradiscal pressure of the adjacent segment, and increase the facet joint stress of the adjacent segment^22^. In this study, a FEM of AdIS was established. After parameter optimization and validation, orthopaedic surgery was simulated, and biomechanical analysis of adjacent segments (proximal and distal) after the surgery was carried out. The results showed that the correction surgery of AdIS would cause ROM to decrease, the Maximum von Mises stress of intervertebral discs at adjacent segments to decrease, and that of facet joints to increase. Therefore, our study demonstrated that the adjacent segment after corrective surgery of AdIS has unique biomechanical characteristics.

### IVD pathogenesis of post-operative ASD in AdIS

Hua et al.^23^ compared the biomechanical changes of adjacent segment degeneration after single-segment or two-segment anterior cervical discectomy and fusion (ACDF) and cervical disc replacement (CDA) through FEA. The results showed that the increase of the pressure in adjacent intervertebral discs may lead to an increased risk of adjacent segment degeneration after ACDF. Zhou et al.^24^ found that the Maximum von Mises stress of IVD at the adjacent segment increased after spinal single segment interbody fusion. However, our research showed different results, the stress of IVD at the post-operative adjacent segment is reduced in AdIS. Wang et al.^25^ found that the decrease of intervertebral disc stress can cause apoptosis of nucleus pulposus cells and an imbalance of the extracellular matrix, leading to the degeneration of the intervertebral disc, through the animal experiment on lumbar vertebrae fixed by pedicle screw rod system. From this, it can be proposed that the reduction of intervertebral disc stress and the imbalance of stress distribution in intervertebral discs may be the biomechanical pathogenesis of adjacent segment degeneration after AdIS corrective surgery.

In addition, our study found that the reduction of Maximum von Mises stress in IVD at the distal adjacent segment was greater than the proximal. Which indicates that the distal adjacent intervertebral disc has a greater probability of accelerated degeneration than the proximal after correction surgery of AdIS. The reduction of the Maximum von Mises stress in IVD at the adjacent segment of Strategy A was smaller than that of Strategy B, which suggests that the probability of adjacent segment degeneration after whole vertebral screw placement is lower. Compared with the upright position, the reduction of the Maximum von Mises stress in IVD at the distal adjacent segment under the other six working conditions is more significant. Which indicates that the hyperactivity of flexion, extension, lateral flexion, and rotation after surgery will accelerate the degeneration of IVD at the adjacent segment.

### FJ pathogenesis of post-operative ASD in AdIS

In a cadaveric study by Li et al.^26^, six C2–C7 cadaveric spine specimens were loaded using four motion modes and a Tecscan pressure test system was used for testing the facet joint pressure. The results showed that the adjacent segment facet joint pressures increased faster after fusion compared with preoperative conditions. In a FEA by Du et al.^27^ to study the mechanical characteristics of adjacent segments after oblique lumbar interbody fusion, an increase in facet joint pressure was observed in the adjacent segment in the oblique lumbar interbody fusion model compared with the healthy model. Similarly, our study confirmed that the Maximum von Mises stress of the adjacent facet joint significantly increased after AdIS corrective surgery. Lu et al.^28^ applied pressure to the caudal vertebra of rats and found that the increase of facet joint stress resulted in a decrease of chondrocytes and the degeneration of facet joints. Li et al.^29^ conducted an animal study to examine the effect of increased articular stress on adjacent segment degeneration, and found that long-term axial loading induces the development of spine hyperalgesia in mice. This result is associated with increased osteoclast activity, aberrant angiogenesis, and nerve invasion into the subchondral bone of the facet joint that stimulates the natural pathological change in facet joint osteoarthritis. Hence, an increase in facet joint stress is involved in the biomechanical pathogenesis of adjacent segment degeneration after AdIS corrective surgery.

In addition, our study confirmed that the increment of Maximum von Mises stress on the L5–S1 facet joint surface was much higher than that on the T5–T6 facet joint surface, which may indicate that the distal adjacent facet joint has a greater probability and degree of accelerated degeneration than the proximal adjacent facet joint after AdIS correction surgery. The increased degree of the Maximum von Mises stress of the facet joint surface in the T5–T6 of Strategy A was smaller than that in Strategy B. This suggests that the probability of proximal adjacent segment facet joint degeneration after pedicle screw implantation in all the segments of both sides was lower than alternate screw instrumentation on both sides. There was no significant difference in the degree of increase of the Maximum von Mises stress of the facet joint surface on the L5–S1 between Strategy A and Strategy B. This suggests that the alternate screw instrumentation on both sides had no impact on the degeneration of facet joint at the distal adjacent segments. Compared with the upright position, the increment of the Maximum von Mises stress of the L5–S1 facet joint surface under the other six working conditions was more significant, which indicates that the postoperative hyperactivity of flexion, extension, lateral flexion, and rotation will accelerate the degeneration of the adjacent facet joint.

### ROM pathogenesis of post-operative ASD in AdIS

Ou et al.^30^ used a goat model to conduct an in vitro experiment to study upper and lower adjacent segment ROM after the fixation of different lumbar spine segments. The results showed that the upper and lower adjacent segment ROMs increased with an increase in the external load. The upper adjacent segment showed significantly greater ROM than the lower adjacent segment ROM in each group. Wang et al.^31^ analysed the biomechanical changes of the lumbar adjacent segment by comparing the biomechanics after the transforaminal lumbar interbody fusion and oblique lumbar interbody fusion surgery. The FEA showed that both transforaminal and oblique lumbar interbody fusion could increase the ROM of the adjacent segment after surgery. However, our research shows different results, where the ROM of the adjacent segment is reduced after AdIS correction surgery. Holewijn et al.^32^, in a prospective gait analysis study of adolescent idiopathic scoliosis patients, showed that the orthopaedic surgery did not induce an increase in distal adjacent segment mobility. A retrospective analysis of an 8.7 year follow-up on the relationship between total disc replacement motion and the development of adjacent segment degeneration proved the prevalence of adjacent segment degeneration after total disc replacement was higher in patients with motions of less than 5°^33^. Another retrospective analysis of 61 patients indicated that the patients who underwent Dynesys stabilization for L4–5 grade 1 spondylolisthesis experienced significantly reduced ROM and a positive correlation of facet degeneration over time postoperatively^34^.

It can be inferred that ROM reduction is another biomechanical cause for the pathogenesis of adjacent segment degeneration after AdIS correction surgery. Moreover, our study found that the ROM decline of the proximal adjacent segment was more significant than that of the distal adjacent segment. This indicates that the proximal adjacent segment has a greater propensity for facet joint degeneration than the distal adjacent segment after AdIS correction surgery. The reduction of the ROM in the adjacent segment of strategy B was smaller than that of strategy A, which suggests that the probability of adjacent segment facet joint degeneration after discontinuous vertebral screw placement is lower. The reduction of ROM of right bending and right rotation after correction surgery was more obvious than that of other operating modes, which indicates that excessive lateral flexion and rotation after surgery will accelerate the adjacent segment degeneration.

There are some limitations to the present study. As the spine structure of AdIS is very complicated, the corresponding FEM had some inherent simplifications and limitations. First, the model could not fully represent the true complexity of spine motion and could only reflect trends in changes of the spine subjected to different loads. Second, in the current FEM, muscles were not simulated, and ligaments were modelled as a one-dimensional nonlinear spring element, which may affect the motion and change in stress of the spine. Third, soft hydrated tissues (intervertebral discs and cartilage at the facet joint) behave as biphasic materials^35^, however, the study modelled soft tissues as elastic bodies, with the bar element used to replace the solid ligament. This simplification of the model has repercussions on the magnitudes of the stress analyses and potential differences across surgical approaches. The situation of the posterior ligament complex in the adjacent segment is important for maintaining segmental stability and reducing the occurrence of postoperative proximal junctional kyphosis. Because it was not a solid model, the posterior ligament stress could not be measured, and therefore another limitation of this study. Fourth, only the correction surgery of posterior pedicle screw fixation and posterior release were simulated. However, severe spinal stiffness is observed in adult scoliosis, which requires auxiliary anterior release to obtain better orthopaedic results. For other correction strategies, such as posterior pedicle screw placement combined with an anterior release, or posterior pedicle screw placement combined with an anterior and posterior release, these results should be explored using a more comprehensive test.

## Conclusions

In conclusion, our study results suggest that the biomechanical characteristics of adjacent segments after correction surgery of AdIS include a decrease in intervertebral disc stress, an increase in facet joint stress, and a decrease in ROM. These characteristics may explain the biomechanical mechanism of adjacent segment degeneration after AdIS correction surgery. The decrease of intervertebral disc stress and ROM in the proximal adjacent segment, and the increase of facet joint stress in the distal adjacent segment was significant. This indicates that the biomechanical mechanism of proximal and distal adjacent segment degeneration is not completely consistent. Additionally, flexion, extension, lateral flexion, and rotation after surgery aggravates the mechanical changes of adjacent segments, suggesting that postoperative hyperactivity can accelerate the degeneration of the adjacent segment. The reduction of the Maximum von Mises stress value of the intervertebral disc in the adjacent segment of “All Vertebral Pedicle Screw Strategy” was smaller than that of “Interval Vertebral Pedicle Screw Strategy”. The increased degree of the Maximum von Mises stress value of the facet joint surface in the T5–T6 of “All Vertebral Pedicle Screw Strategy” was smaller than that in the “Interval Vertebral Pedicle Screw Strategy”. The reduction of ROM in the adjacent segment of the “Interval Vertebral Pedicle Screw Strategy” was smaller than that of the “All Vertebral Pedicle Screw Strategy”. This suggests that the “All Vertebral Pedicle Screw Strategy” is better than “Interval Vertebral Pedicle Screw Strategy” in reducing the incidence of adjacent segment degeneration after surgery to correct for AdIS.

## Data Availability

The datasets used and/or analysed during the current study are available from the corresponding author on reasonable request.

## Abbreviations

ASD: Adjacent segment degeneration
AdIS: Adult idiopathic scoliosis
IVD: Intervertebral disc
FJ: Facet joint
ROM: Range of motion
CT: Computed tomography
FEA: Finite element analysis
FEM: Finite element model
